# Detection of *Toxoplasma gondii* in retail meat samples in Scotland

**DOI:** 10.1016/j.fawpar.2020.e00086

**Published:** 2020-06-12

**Authors:** Jacqueline Plaza, Filip Dámek, Isabelle Villena, Elisabeth A. Innes, Frank Katzer, Clare M. Hamilton

**Affiliations:** a Moredun Research Institute, Pentlands Science Park, Midlothian EH26 0PZ, UK; b Laboratory of Parasitology, EA 7510, University of Reims Champagne-Ardenne and National Reference Centre on Toxoplasmosis, Hospital University Centre of Reims, France

**Keywords:** *Toxoplasma gondii*, Meat, Venison, PCR, Antibodies, Genotyping

## Abstract

*Toxoplasma gondii* is a globally important zoonotic parasite ranked as one of the most significant causes of disease burden among the major foodborne pathogens. Consumption of undercooked meat is a well-known risk factor for infection so the aim of this study was to investigate the presence of *T. gondii* in meat samples from retail outlets in Scotland. In Sampling Period 1, 300 meat samples (39 beef, 21 chicken, 87 lamb, 71 pork and 82 venison) were purchased from butchers', farmers' markets, farm shops and supermarkets, and in Sampling Period 2, 67 pure venison samples only were purchased from farmers' markets, farm shops and supermarkets. DNA was extracted and screened for *T. gondii* using a quantitative PCR targeting the 529 bp repeat element, and any positive samples were genotyped using PCR-RFLP targeting 10 markers. Meat juice was screened for *T. gondii* antibodies using a commercial ELISA or modified agglutination assay. *Toxoplasma gondii* DNA was detected in 0/39 (0%) beef samples, 1/21 (4.8%) chicken samples, 6/87 (6.9%) lamb samples, 3/71 (4.2%) pork samples and 29/82 (35.4%; Sampling Period 1) and 19/67 (28.4%; Sampling Period 2) venison samples. Partial PCR-RFLP genotyping revealed both clonal and non-clonal genotypes. Antibodies to *T. gondii* were detected in the meat juice of 2/38 (5.3%) beef samples, 3/21 (14.3%) chicken samples, 14/85 (16.5%) lamb samples, 2/68 (2.9%) pork samples and 11/78 (14.1%; Sampling Period 1) and 8/50 (16%; Sampling Period 2) venison samples. This is the first study to report the presence of *T. gondii* in retail meat products in Scotland and has highlighted venison as a potentially high risk meat. Further work is required to determine viability of parasites in this particular meat product.

## Highlights

• *Toxoplasma gondii* DNA was detected in pork, lamb, chicken and venison products.
• None of the beef samples tested were positive for *T. gondii* DNA.
• Venison could be a high-risk meat for *T. gondii* infection.
• Non-clonal genotypes may be present in venison products in Scotland.

## 1 Introduction

*Toxoplasma gondii* is a zoonotic parasite of global importance. Humans can become infected with the parasite via ingestion of oocysts (shed in cat faeces) directly from the soil or in contaminated food or water; via ingestion of tissue cysts in undercooked/raw infected meat; or vertically from mother to baby during a primary infection in pregnancy. Symptoms of toxoplasmosis in immune competent people are mostly mild and self-limiting; however, immune compromised people can suffer severe or life-threatening disease and acute infection during pregnancy may result in miscarriage or devastating congenital defects (Dubey, 2010).

In the UK, an average of 365 cases of toxoplasmosis are clinically diagnosed in England and Wales each year and an average of 33 cases are clinically diagnosed annually in Scotland (Halsby et al., 2014; HPS, 2018). However, given the lack of pathognomonic signs in the majority of infections and the fact that the disease is not notifiable, these figures are likely to be an underestimate of the true incidence of toxoplasmosis. In a recent study of over 1400 blood donors in Scotland, 13.2% had antibodies to *T. gondii* (Burrells et al., 2016)*.* In the same study, *T. gondii* DNA was also detected in 17.9% of brain tissue deposited at the Medical Research Council Sudden Death Brain Bank. In both study groups, prevalence of *T. gondii* increased with age suggesting an important role for acquired infection.

Foodborne transmission of *T. gondii* is an important route of infection and consumption of undercooked/raw meat is known to be a significant risk factor (Belluco et al., 2018). Due to the severe sequealae of infection which may persist for the lifetime of the host, the disease burden of toxoplasmosis can be high (Scallan et al., 2015). Recent studies in the USA and the Netherlands ranked *T. gondii* as the second and third highest cause of disease burden among the major foodborne pathogens, respectively (Scallan et al., 2015; EFSA, 2018). Despite bodies such as the Food Standards Agency and European Food Safety Authority highlighting the need for identifying the role of different meat products in foodborne toxoplasmosis, there have been few studies examining the presence of *T. gondii* in commercial meat products (Warnekulasuriya et al., 1998, Dubey et al., 2005).

The aim of the present work, therefore, was to investigate the presence of *T. gondii* in different meat products available for human consumption from retail outlets in Scotland.

## 2 Materials and methods

### 2.1 Sample collection

Convenience meat samples were collected over two periods. Initially, 300 meat samples (39 beef, 21 chicken, 87 lamb, 71 pork and 82 venison) were purchased from different retail outlets between April and November 2017 (Sampling Period 1; Table 1). Of the 300 samples, 31 were purchased from butchers' shops, 163 were purchased from farmers' markets or farm shops, and 106 were purchased from supermarkets. All samples were purchased fresh (not frozen) and were pre-packaged except those purchased at butcher shops which were packaged upon purchase. Different cuts of meat were purchased for each meat type on a convenience basis (Table 1). The rearing conditions of the animals was not always available but where it was: 22 out of 39 beef samples were from pasture-reared animals, 10 out of 21 chicken samples were from outdoor-reared animals, 20 out of 71 pork samples were from outdoor-reared animals and all lamb samples were from animals reared in the UK so were assumed to be outdoor-reared (Supplementary Table 1). Of the 82 venison samples, 46 were known to be from wild deer, 23 were from farmed deer, 2 were from a mix of wild and farmed deer and for 11 samples the origin was not specified (Supplementary Table 1). As some of the venison products in Sampling Period 1 also contained pork, a second collection (Sampling Period 2) was carried out in which 67 samples of pure venison only were purchased (fresh, not frozen) between June and August 2018 (Table 1). Of these, 50 were purchased at farmers' markets or farm shops and 17 were purchased from supermarkets. Out of 67 samples, 28 were from wild deer and 39 were from farmed deer (Supplementary Table 1).

**Table 1:** Cuts of meat sampled.

Meat Type	Cut of meat	No. purchased
*Sampling Period 1:*		
Beef	Ground meat^a^ (sausages, minced, burgers, meatballs) Steak (shoulder, rump, fillet) Stewing meat TOTAL	24 10 5 39
Chicken	Breast Drumstick Offal (liver) TOTAL	19 1 1 21
Lamb	Steaks (fillet, leg) Ground meat^b^ (sausages, grillsteaks, minced, burgers) Meat on the bone (chops, shank) Stewing meat Offal (heart, kidney, liver) TOTAL	29 15 13 6 24 87
Pork	Ground meat^c^ (sausages, burgers, minced) Steak (fillet, loin, leg, shoulder) Stewing meat Bacon and loin medallions Chops Offal (heart, liver) TOTAL	35 21 7 4 2 2 71
Venison	Ground meat^d^ (sausages, grillsteaks, burgers, meatballs, mince) Stewing meat Steak (haunch, striploin) Offal (liver, kidney) Other (frying meat) TOTAL	58 14 5 3 2 82

*Sampling Period 2:*		
Venison	Steak (haunch, sirloin) Ground meat^e^ (burgers and mince) Stewing meat Other (meat for frying) TOTAL	28 21 12 6 67

a Samples contained beef only;

b Samples contained lamb only;

c Samples contained pork only;

d 43 out of 58 samples also contained pork;

e Samples contained venison only.

Although samples were purchased over an 8-month period (Sampling Period 1) and a 3-month period (Sampling Period 2) from a range of different retail outlets, it is possible that some of the samples originated from the same animal. However, the purpose of the study was not to determine the prevalence of *T. gondii* in individual animals but to determine the incidence in meat products for sale to the general public.

### 2.2 Sample processing and testing

Fifty grams per meat sample were digested with acid-pepsin and DNA was extracted from 2 ml homogenised pellet, as previously described (Hamilton et al., 2015). Meat juice (fluid within the meat packaging) was collected from fresh samples where possible as previously described (Hamilton et al., 2015). Where none was available, juice was collected following freezing and thawing of the remaining meat sample once 50 g had been taken for DNA extraction.

DNA from each sample was screened for *T. gondii* DNA using a quantitative PCR (qPCR) targeting the 529 bp repeat element, as previously described (Hamilton et al., 2015). Any samples which were positive by qPCR were electrophoresed on a 3% agarose gel incorporating Biotium GelRed™ (Cambridge Bioscience Ltd., U·K) to confirm the size of amplicons. Genotyping was attempted on qPCR-positive samples using a multiplex nested PCR-RFLP targeting 10 genetic markers, including SAG1, SAG2 (5′-3′ SAG2 and alt.SAG2), SAG3, BTUB, GRA6, c22–8, c29–2, L358, PK1 and Apico. PCR-RFLP conditions for all markers were carried out as previously described (Hamilton et al., 2015).

Meat juice collected from beef, lamb, pork and venison were screened for antibodies to *T. gondii* using an indirect ELISA (ID Screen® Toxoplasmosis Indirect Multi-species, IDvet, Montpellier, France) according to the manufacturer's instructions. Meat juice collected from chicken samples were sent to the Toxoplasma National Reference Centre (Reims, France) to be screened using a modified agglutination test as previously described (Halos et al., 2010). As the MAT has previously been shown to have lower diagnostic specificity for fluids collected from muscle tissue such as chicken breast (Schares et al., 2018), we used a higher cut-off of 1:20 to reduce the number of false positive results.

### 2.3 Statistical analysis

Statistical analysis was carried out using Minitab Statistical Software (version 17). Since two detection methods (serological and molecular) were used to test the meat samples for the presence of *T. gondii*, the level of agreement between the tests was investigated using Cohen's kappa coefficient (Landis and Koch, 1977).

## 3 Results

Of the 300 meat samples purchased for testing in Sampling Period 1, 39 (13.0%) were found to be positive for *T. gondii* DNA (Table 2). Specifically, *T. gondii* DNA was detected in 1 out of 21 (4.8%) chicken samples, 6 out of 87 (6.9%) lamb samples, 3 out of 71 (4.2%) pork samples and 29 out of 82 (35.4%) venison samples (Table 2). None of the 39 beef samples were positive by qPCR. Of the 67 venison samples purchased in Sampling Period 2, 19 (28.4%) were positive for *T. gondii* DNA (Table 2). The number of positive samples for the different rearing conditions and by the different cuts of meat are reported in Supplementary Tables 1 and 2, respectively. Partial genotyping was obtained for only 3 out of the 58 qPCR-positive meat samples (from both sampling periods) due to lack of amplification at the single-copy markers. Of the 3 samples genotyped, all of which were from venison, amplification could only be achieved at 3 markers for one sample (venison sample 1) and 4 markers for two samples (venison samples 2 and 3). Venison sample 1 had Type I alleles at markers SAG2 (5′-3′), L358 and Apico. Venison sample 2 had Type II alleles at markers SAG2 (5′-3′ and alt. SAG2), SAG3 and BTUB. Venison sample 3 had a Type I allele at GRA6, Type II alleles at C22–8 and L358, and a Type III allele at SAG3 suggesting a non-clonal genotype (atypical).

**Table 2:** Detection of *Toxoplasma gondii* in meat samples by qPCR and serology.

Meat product	No. tested by qPCR	No. positive by qPCR (%)	No. tested by serology^a^	No. positive by serology (%)	No. positive by PCR and/or serology (%)
*Sampling period 1:*					
Beef Chicken Lamb Pork Venison TOTAL	39 21 87 71 82 300	0 (0%) 1 (4.8%) 6 (6.9%) 3 (4.2%) 29 (35.4%) 39 (13.0%)	38 21 85 68 78 290	2 (5.3%) 3 (14.3%) 14 (16.5%) 2 (2.9%) 11 (14.1%) 32 (11.1%)	2 (5.1%) 4 (19.0%) 17 (19.5%) 4 (5.6%) 30 (36.6%) 57 (19.0%)

*Sampling period 2:*					
Venison	67	19 (28.4%)	50	8 (16.0%)	20 (29.9%)

a Beef, Lamb, Pork and Venison meat juices were all tested by ELISA whereas chicken meat juice was tested by MAT.

Meat juice could not be collected from all meat samples (Table 2). In Sampling Period 1, fresh meat juice could only be collected from 193 samples and juice from frozen and thawed samples was collected from 97 samples (no juice could be collected from 10 samples despite numerous freeze/thaw cycles). Antibodies to *T. gondii* were detected in 32 out of 290 (11.0%) meat juice samples tested in Sampling Period 1 (Table 2). Specifically, antibodies were detected in 2 out of 38 (5.3%) beef juice samples, 3 out of 21 (14.3%) chicken juice samples, 14 out of 85 (16.5%) lamb juice samples, 2 out of 68 (2.9%) pork juice samples and 11 out of 78 (14.1%) venison juice samples. In Sampling Period 2, fresh meat juice could only be collected from 28 samples and juice from frozen and thawed samples was collected from 22 samples (no juice could be collected from 17 samples). *T. gondii* antibodies were detected in 8 out of 50 (16%) venison juice samples tested in Sampling Period 2 (Table 2). The number of serology-positive samples for the different rearing conditions and by the different cuts of meat are reported in Supplementary Tables 1 and 2, respectively.

Overall, 21 samples tested positive by serology (ELISA or MAT) but were negative by qPCR, and 34 samples tested positive by qPCR but were negative by serology. Cohen's kappa coefficient demonstrated only a fair agreement between the tests (κ = 0.348).

## 4 Discussion

The consumption of undercooked or raw meat is a significant risk factor for infection with *T. gondii*. Despite this, few data are currently available on the risk for consumers from retail meat in the UK. In the present study, *T. gondii* DNA was detected in chicken, lamb, pork and venison products purchased for human consumption from different retail outlets in Scotland. Of particular note was the incidence of *T. gondii* in venison which was much higher than any of the other meat types tested. Although some of the venison products in the first sampling period also contained pork meat, the prevalence of *T. gondii* in pure venison samples in the second sampling period was similar indicating that the source of *T. gondii* was most likely the venison, particularly given the low prevalence in pork products overall.

Consumption of undercooked or raw game meat has been identified as the source of *T. gondii* infection in a number of cases of toxoplasmosis (Sacks et al., 1983, McDonald et al., 1990, Ross et al., 2001, England et al., 2019) and very recently there was an outbreak of acute toxoplasmosis in a group of Canadian deer hunters who had consumed undercooked venison steak on a hunting trip to the USA (Gaulin et al., 2020). Venison is seen as a healthy meat choice and in the UK as a whole the venison market is worth an estimated £100 million (Scotland Food and Drink, 2018). As part of Scotland Food and Drink Ambition 2030, there is a significant planned expansion of the farmed venison sector with an emphasis on making venison the main meat of choice for consumers. As it is common (and sometimes recommended on packaging) to consume venison undercooked, this meat could present a potentially significant source of foodborne toxoplasmosis.

The incidence of *T. gondii* detected in meat products in the present study is lower than those reported in a worldwide systemic review and meta-regression analysis on studies reporting the direct detection of *T. gondii* in meat products (Belluco et al., 2016). In this, they reported pooled prevalences of 14.7% in sheep products, 12.3% in pig products and 2.6% in cattle products. These higher prevalences may be a reflection of the type of samples tested as the majority of samples in the analysis were liver, muscle, brain, heart or diaphragm which are known to be predilection sites of the parasite. The lack of detection of *T. gondii* DNA in beef products in the present study is similar to results of other studies (Opsteegh et al., 2011b). Cattle were thought to be able to clear *T. gondii* infections (Burrells et al., 2018) and as such have not been seen as an important source of foodborne toxoplasmosis. However, recent studies have highlighted beef as a high risk meat due it being commonly consumed undercooked or raw (e.g. steak tartare) and therefore the risk from this meat should not be ignored (Opsteegh et al., 2011a; Belluco et al., 2018). In a recent study in Australia, 68% of lamb mincemeat samples purchased at the supermarket were positive for *T. gondii* by PCR highlighting lamb as a potentially high risk meat (Dawson et al., 2019).

It should be noted that the incidence of *T. gondii* detected in meat products in the present study does not reflect the prevalence of *T. gondii* in these food animals. It is possible that some of the products originated from the same animal or were a combination of animals (e.g. ground meat). However, the aim of this study was not to determine the prevalence of *T. gondii* in food animals but instead to determine the incidence in meat products and thus the potential risk to consumers.

The seroprevalence of *T. gondii* in livestock varies widely depending on geographical region, serological test, age of animal and farm management system (Dubey, 2010). Few serological studies have been conducted in the UK. In the present study, antibodies to *T. gondii* were detected in the meat juice of lamb, venison, chicken, beef and pork. Although these results do not directly represent the seroprevalence in these food animals, the results are similar to previous studies in the UK which have reported seroprevalences of 37.3% in 1-year old sheep (Katzer et al., 2011), 32.5% in red deer (Williamson et al., 1980), 11.8% in cattle (Opsteegh et al., 2019) and 3.6% in pigs (Limon et al., 2017). The lack of concordance between serology and direct detection of parasites has been reported previously (Opsteegh et al., 2016; Halos et al., 2010). In the present study, there were samples of all meat types which were positive by serology yet negative by qPCR. This may reflect the size of meat sample processed, the portion of meat tested (an edible portion rather than a predilection site) or the inhomogeneous distribution of tissue cysts. Surprisingly, 18 venison samples which were positive by qPCR were negative by serology. The meat juice samples in this study were screened using a commercial ELISA which, although is suitable for ruminants, has not been specifically validated for deer. The discrepancy may also indicate that the venison originated from deer harbouring a chronic infection and thus although they had cysts in their tissues, their antibody response may have waned (Williamson et al., 1980).

It is of note that out of three DNA isolates, which were partially genotyped in this study, only one had Type II alleles at all the amplified markers and the others had Type I or a mix of alleles. Strains containing Type I or atypical alleles are more pathogenic or more likely to cause severe disease than other isolates (Xiao and Yolken, 2015) and have been associated with cases of clinical human toxoplasmosis in England and Wales (Aspinall et al., 2003) and a case of re-infection of an immune competent patient with devastating consequences (Elbez-Rubinstein et al., 2009). The presence of *T. gondii* with Type I alleles in a meat product which is commonly consumed undercooked, such as venison, could pose a potentially significant public health problem. Type I alleles have also been reported in *T. gondii* DNA isolates from wildlife in Scotland (Burrells et al., 2013) suggesting that other clonal or atypical strains may dominate in the environment.

In conclusion, this is the first study to report the presence of *T. gondii* in retail meat products in Scotland and has highlighted venison as a potentially high risk meat. This data could be used to inform quantitative microbial risk assessments of foodborne toxoplasmosis in Scotland. Further work is underway with additional fresh samples of venison to determine the viability of *T. gondii* in these meat products to truly assess the foodborne risk. Given the difficulty in controlling transmission in food animals, thorough cooking or freezing of meat is currently the best option for reducing human infection (Dubey, 2010). With an ever increasing demand for organic or outdoor-reared meat, there comes an increased risk of infection with *T. gondii* as animals raised in this way are more likely to be seropositive and, therefore, potentially harbouring tissue cysts (Stelzer et al., 2019). Experimental vaccination of pigs (Burrells et al., 2015) and lambs (Katzer et al., 2014) with the S48 strain of *T. gondii* demonstrated a reduction in the number of viable tissue cysts following oocyst challenge resulting in safer meat for consumption; however, there is currently no incentive for farmers to vaccinate their animals purely for food safety. Also, animals would need to be vaccinated shortly after birth to avoid natural infection with *T. gondii* from the environment.

## Declaration of competing interest

The authors declare that they have no known competing financial interests or personal relationships that could have appeared to influence the work reported in this paper.

## Appendix A Supplementary data

Supplementary tables Image 1
