# Supplementary material for: Detection of Toxoplasma gondii in retail meat samples in Scotland
Source: Food Waterborne Parasitol. 2020 Jun 12;20:e00086. doi: 10.1016/j.fawpar.2020.e00086 (PMC7303546; doi:10.1016/j.fawpar.2020.e00086)
Supplement: Supplementary file 1 — Supplementary tables [file mmc1.docx]

**Supplementary Table 1.** Detection of *T. gondii* in meat samples from animals raised under different rearing conditions

| **Meat Sample and rearing condition** | **No. tested by PCR** | **No. pos by PCR** | **No. tested by serology** | **No. pos by serology** | **No. pos by PCR and/or serology** |
| --- | --- | --- | --- | --- | --- |
| *Sampling Period 1*  **Beef**  Outdoor, pasture-fed  Unknown  **Chicken**  Outdoor, organic  Unknown  **Lamb**  Outdoor, grass-fed  **Pork**  Outdoor  Indoor  Unknown  **Venison**  Wild  Farmed  Mix of wild and farmed venison  Unknown  *Sampling Period 2*  **Venison**  Wild  Farmed | 22  17  10  11  87  20  10  41  46^a^  23^b^  2^b^  11^c^  28^b^  39^b^ | 0  0  0  1  6  2  0  3  23  3  0  3  8  11 | 21  17  10  11  85  20  10  38  42  23  2  11  17  33 | 1  1  2  1  14  0  0  2  9  2  0  0  3  5 | 1  1  2  4  17  2  0  2  23  4  0  3  9  11 |

^a^28 products also contained pork; ^b^Products were pure venison only; ^c^7 products also contained pork

**Supplementary Table 2.** Detection of *T. gondii* in different cuts of meat sampled

| **Meat Type** | **Cut of meat** | **No. tested by PCR** | **No. pos by PCR** | **No. tested by serology** | **No. pos by serology** | **No. pos by PCR and/or serology** |
| --- | --- | --- | --- | --- | --- | --- |
| *Sampling Period 1:*  **Beef**  **Chicken**  **Lamb**  **Pork**  **Venison**  *Sampling Period 2:*  **Venison** | Ground meat^a^ (sausages, minced, burgers, meatballs)  Steak (shoulder, rump, fillet)  Stewing meat  Breast  Drumstick  Offal (liver)  Steaks (fillet, leg)  Ground meat^b^ (sausages, grillsteaks, minced, burgers)  Meat on the bone (chops, shank)  Stewing meat  Offal (heart, kidney, liver)  Ground meat^c^ (sausages, burgers, minced)  Steak (fillet, loin, leg, shoulder)  Stewing meat  Bacon and loin medallions  Chops  Offal (heart, liver)  Ground meat^d^ (sausages, grillsteaks, burgers, meatballs, mince)  Stewing meat  Steak (haunch, striploin)  Offal (liver, kidney)  Other (frying meat)  Steak (haunch, sirloin)  Ground meat^e^ (burgers and mince)  Stewing meat  Other (meat for frying) | 24  10  5  19  1  1  29  15  13  6  24  35  21  7  4  2  2  58  14  5  3  2  28  21  12  6 | 0  0  0  1  0  0  1  2  2  0  1  2  0  0  0  1  0  25  1  2  0  1  8  5  6  0 | 23  10  5  19  1  1  29  13  13  6  24  32  21  7  4  2  2  54  14  5  3  2  27  11  11  1 | 2  0  0  3  0  0  2  2  3  1  5  1  0  0  0  0  1  8  1  2  0  0  2  1  5  0 | 2  0  0  4  0  0  3  3  4  1  6  2  0  0  0  1  1  26  1  2  0  1  8  5  7  0 |

^a^Samples contained ground beef only; ^b^Samples contained ground lamb only; ^c^Samples contained ground pork only; ^d^43 out of 58 samples also contained ground pork; ^e^Samples contained ground venison only.
